# Metabolite database for root, tuber, and banana crops to facilitate modern breeding in understudied crops

**DOI:** 10.1111/tpj.14649

**Published:** 2020-01-22

**Authors:** Elliott J. Price, Margit Drapal, Laura Perez‐Fons, Delphine Amah, Ranjana Bhattacharjee, Bettina Heider, Mathieu Rouard, Rony Swennen, Luis Augusto Becerra Lopez‐Lavalle, Paul D. Fraser

**Affiliations:** ^1^ Royal Holloway University of London, Surrey TW20 0EX Egham United Kingdom; ^2^ International Institute of Tropical Agriculture PMB 5320 Ibadan Nigeria; ^3^ International Potato Center, La Molina CP 1558 Lima Peru; ^4^ Bioversity International Parc Scientifique Agropolis II 34397 Montpellier France; ^5^ Laboratory of Tropical Crop Improvement Division of Crop Biotechnics KU Leuven B‐3001 Leuven Belgium; ^6^ Bioversity International Willem De Croylaan 42 B‐3001 Leuven Belgium; ^7^ International Institute of Tropical Agriculture. C/0 The Nelson Mandela African Institution of Science and Technology P.O. Box 44 Arusha Tanzania; ^8^ International Center for Tropical Agriculture CP 763537 Cali Colombia; ^9^ Present address: Masaryk University Brno‐Bohunice 625 00 Czech Republic

**Keywords:** Banana and plantain (*Musa* spp.), cassava (*Manihot esculenta*), potato (*Solanum tuberosum*), sweet potato (*Ipomoea batatas*), yam (*Dioscorea* spp.), metabolomics, genebanks, modern breeding

## Abstract

Roots, tubers, and bananas (RTB) are vital staples for food security in the world's poorest nations. A major constraint to current RTB breeding programmes is limited knowledge on the available diversity due to lack of efficient germplasm characterization and structure. In recent years large‐scale efforts have begun to elucidate the genetic and phenotypic diversity of germplasm collections and populations and, yet, biochemical measurements have often been overlooked despite metabolite composition being directly associated with agronomic and consumer traits. Here we present a compound database and concentration range for metabolites detected in the major RTB crops: banana (*Musa* spp.), cassava (*Manihot esculenta*), potato (*Solanum tuberosum*), sweet potato (*Ipomoea batatas*), and yam (*Dioscorea* spp*.*), following metabolomics‐based diversity screening of global collections held within the CGIAR institutes. The dataset including 711 chemical features provides a valuable resource regarding the comparative biochemical composition of each RTB crop and highlights the potential diversity available for incorporation into crop improvement programmes. Particularly, the tropical crops cassava, sweet potato and banana displayed more complex compositional metabolite profiles with representations of up to 22 chemical classes (unknowns excluded) than that of potato, for which only metabolites from 10 chemical classes were detected. Additionally, over 20% of biochemical signatures remained unidentified for every crop analyzed. Integration of metabolomics with the on‐going genomic and phenotypic studies will enhance ’omics‐wide associations of molecular signatures with agronomic and consumer traits via easily quantifiable biochemical markers to aid gene discovery and functional characterization.

## Significance Statement

A metabolite‐specific database cataloguing the biochemical diversity within and between root, tuber, and banana (RTB) crops has been compiled from profiling thousands of accessions. The database records the extent of metabolite concentrations available in screened germplasm of each RTB crop and therefore can be used to set breeding targets. This information aids in crop breeding programmes to improve the livelihoods for more than two billion people reliant on RTB crops.

**Highlights**

- Root, tuber and banana (RTB) crops are consumed by over 2 billion people.
- A comparative metabolomics workflow is applied to RTB crops.
- Biochemical diversity of understudied species was captured and is a freely available data resource.
- Potential application in breeding programmes, for example bio‐fortification, disease resistance mechanisms, and stress tolerance.
- Integration into multiomic workflows.

## Introduction

### Importance of RTB crops

**Figure 1:**
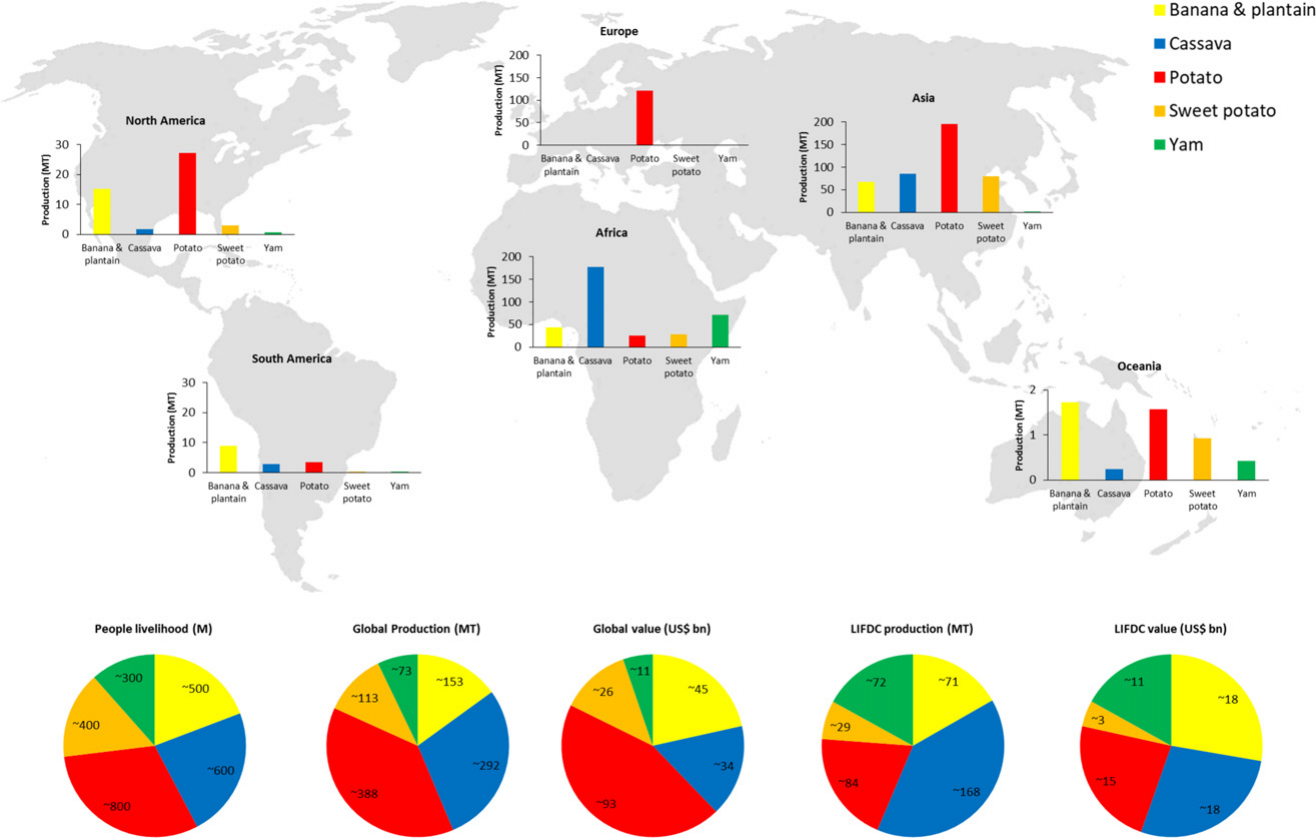
Production of root, tuber, and banana (RTB) crops. Global and continental production of RTB crops highlights their importance as a staple food and livelihood for billions of people especially in Low Income Food Deficit Countries (LIFDCs). Data taken from FAOSTAT (production data for 2017, value data for 2016) (Food and Agriculture Organization of the United Nations, 2019). World map image modified from www.freevectormaps.com

The annual global production of root, tuber, and banana (RTB) crops exceeds 1000 million tonnes (Food and Agriculture Organization of the United Nations, 2019) and feeds over 2 billion people worldwide (Scott *et al.*, 2000) (Figure 1). RTBs are especially vital in the least developed countries where they provide ≥15% of daily calories and are a source of economic subsistence to over 750 million people (Kennedy *et al.*, 2019). In Africa, the production of RTBs exceeds that for all other staples combined (Sanginga, 2015) and are the most important crops for direct human consumption. Over 30 000 RTB crop accessions are currently held in the genebanks of four CGIAR institutes with many further accessions in national and regional collections, representing the diversity currently available for breeding (Tay, 2013). Whilst the RTB crops are cited to have high yield potential (especially regarding calories per hectare production) when compared with other staples (cereals), the extent of diversity available for breeding cannot be capitalized upon due to limited knowledge on the biological potential of these accessions. In addition to the dearth of genetic resources, basic characterization such as phenotypic and agronomic traits, including growth and yield parameters, are scarce for a large proportion of accessions. Consequently, insufficient germplasm characterization and evaluation has hindered the exploitation of the available diversity within breeding programmes (Jansky *et al.*, 2015). Depending on the RTB crop three factors have contributed, to a varying degree, to the current situation: (i) poor or under‐representation of crop wild relatives in germplasm collections (Castañeda‐Álvarez *et al.*, 2016); (ii) high levels of accession duplication and misidentifications in the collections, particularly prevalent in clonal crop collections (yam up to 30% (Girma *et al.*, 2012), potato varies from *c. *4.5 % (Ellis *et al.*, 2018) to *c. *75 % (Huamán *et al.*, 2000) across different subsets); and (iii) the poorly recorded assessment of germplasm diversity, which is especially complex in RTB crops due to crop wild gene flow via ennoblement, hybridization from overlapping natural and cultivation habitats, and genetic assimilation from vegetative propagation (Scarcelli *et al.*, 2017).

In recent years many large‐scale efforts have sought to further understand these crops using genome sequences (Xu *et al.*, 2011; D'Hont *et al.*, 2012; Wang *et al.*, 2014; Tamiru *et al.*, 2017; Yang *et al.*, 2017; Li *et al.*, 2019) and genome diversity studies (Bredeson *et al.*, 2016; Hardigan *et al.*, 2017; Nyine *et al.*, 2017; Christelová *et al.*, 2017; Muñoz‐Rodríguez *et al.*, 2018; Němečková *et al.*, 2018), genetic selection (Wolfe *et al.*, 2016), molecular markers (QTLs) (Monden and Tahara, 2017; Kim *et al.*, 2017; Sharma and Bryan, 2017), and comparative transcriptome resources (Kundapura Venkataramana *et al.*, 2015; Sarah *et al.*, 2017; van Wesemael *et al.*, 2019; Cenci *et al.*, 2019) widely developed alongside morphologic, agronomic and phenotypic classifications (Oliveira *et al.*, 2015; Rahajeng and Rahayuningsih, 2017; Dépigny *et al.*, 2018; Girma *et al.*, 2018; van Wesemael *et al.*, 2019). The progress of the CGIAR Research Program on Roots, Tubers and Bananas (www.rtb.cgiar.org), applying genomics‐assisted breeding to RTBs, has recently been reviewed (Friedmann *et al.*, 2018). Although typically in the early stages, the authors noted that success will be dependent upon the quality of phenotypic characterization.

### Why metabolomics in breeding?

Agronomic and consumer traits can often be directly associated with metabolite composition (Bino *et al.*, 2004), which favours the use of metabolomics to generate measurable biochemical signatures for characterization. Metabolomics approaches can provide a standalone technique when genetic mechanisms are not well understood (Price *et al.*, 2017), as evident in RTB crops. Phenotypic evaluation of materials is required multiple times along the breeding pipeline and integration of metabolomics into current practices is advocated to greatly shorten the development time of new varieties, reduce costs, and provide unbiased phenotypic profiles for validation of genetic parameters (Fernie and Schauer, 2009), and has the potential of being a powerful approach for future precision breeding (Zivy *et al.*, 2015).

Various different metabolomics approaches can be undertaken, generally encompassing untargeted metabolite profiling including broad‐scale relative quantification of known and unknown metabolites and targeted profiling and absolute quantification of identified metabolites. As the accuracy of identification and quantification increases, so does the time required for analysis. Through integration with other ’omics to associate genotype with phenotype, the regulation of agronomic/ phenotypic traits (phenomics) at the genetic (genomics, epigenomics), transcriptional (transcriptomics), translational (proteomic) and metabolic level (metabolomics) can be dissected in a holistic systems biology manner to enhance the understanding of crop development and its responses to biotic and abiotic changes. The development of bioinformatics tools and resources has rapidly progressed alongside ’omics technologies to facilitate the integration and management of these large and complex datasets. However, the interpretation of integrated datasets is complex, requiring expertise and collaboration across many scientific fields, and remains the major challenge for multiomics investigations (Pinu *et al.*, 2019; Misra *et al.*, 2019). This system biology approach has already been applied to model crops such as tomato, rice, and wheat, in which metabolomics analyses have provided a richness of resources (Grennan, 2009; Perez‐Fons *et al.*, 2014) available to integrate with genetic breeding approaches. These resources rapidly accelerated progress for identifying trait markers (Schwahn *et al.*, 2014; Li *et al.*, 2016a; Sprenger *et al.*, 2018), elucidation of biosynthetic pathways contributing to traits (Schwahn *et al.*, 2014; Daygon *et al.*, 2017), and validation of genetic/ metabolic prediction (Wei *et al.*, 2018). For example, integrating genetic and metabolite markers for phenotypic traits of wheat has provided more robust signatures than either alone (Ward *et al.*, 2015), and both were equally predictive for complex traits (Riedelsheimer *et al.*, 2012).

Furthermore, metabolite markers are inherently affected by environmental factors and can provide more precise measures for crop trait variation compared with genetic markers. Metabolite markers can be stably inherited (Chan *et al.*, 2010) and, as such, the metabolome can be viewed in an analogous manner to the epigenome, acting as a dynamic yet conserved network comprised from genetic and environmental influence. Consequently, when performing comparative analyses of crop growth under different environments, quantifying the contributions of biochemical signatures towards phenotype is often simpler than for genetic markers, especially in highly heterozygous crops, like RTBs. This gives rise to the potential to generate chemotype core collections (CCC) for use in breeding, in which material selection is based on fixation of a complement of biochemical signatures that could confer the desired characteristics more robust to environmental variation. This is contrary to genotypic core collections, in which breeding tries to fix gene variants that can then often harbour different traits under different environments. Furthermore, increased trait stability of CCCs would provide a suitable base for comparative GxE (Genotype × Environment) studies to elucidate environmental effects on crop production (Xu, 2016). CCCs would therefore complement genotypic core collections to facilitate localized precision breeding in the future.

Despite these advantages, the deployment of enhanced cultivars directly from metabolomics‐directed breeding is still limited, largely based on the slow uptake by breeders and the limited access to this technology, with the field still being listed as prospective but with the potential to be game‐changing for future agricultural practice (Kumar *et al.*, 2017).

### Prospective societal impact

Given the role that RTB crops play in the livelihoods of millions of people in the least developed nations, improvement is paramount. On the whole, RTBs are primarily grown through small‐holder farms with a large proportion of child and female labour and, therefore, the crops hold extreme importance for the most vulnerable portions of society.

**Figure 2:**
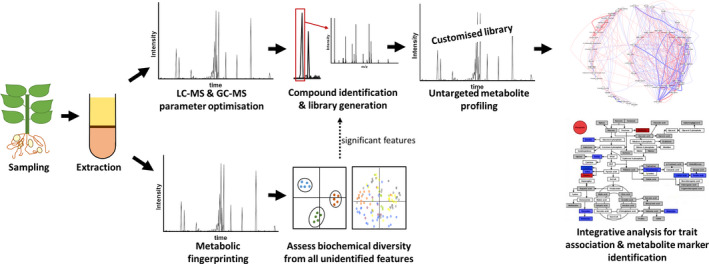
Workflow of metabolomics analysis established to screen biochemical diversity of root, tuber, and banana crops. The use of numerous and complementary analytical platforms provides a more comprehensive coverage of the metabolome; customized libraries specific for each crop reduce matrix effects. Metabolic fingerprint analysis typically takes *c. *20 min per sample and generates *c. *10 000 features, with data analysis being *c. *1 h per 100 samples. Library creation is on‐going but requires *c. *20 h per crop before implementing automation, inclusive of machine time. Untargeted metabolite profiling takes *c. *60 min per sample per analytical platform and data analysis plus manual curation takes *c. *10 h per 100 samples. Example statistical visualizations created using SIMCA‐P (Umetrics), Metscape (Basu *et al.*, 2017) in Cytoscape (Shannon, 2003), and an in‐house pathway mapper, Biosynlab (Royal Holloway University of London, UK).

Increasing the precision and speed of phenotyping during the breeding ladder (Figure 2) would enable faster crop improvements and, therefore, a multitude of benefits: (i) enhanced agronomic, breeding efficiency and consumer traits (e.g. increased yields, increased flowering, reduced dormancy and bio‐fortification) to tackle food insecurity and malnutrition, which are more prevalent in RTB growing regions; (ii) decreased fertilizer inputs and improved pest and disease resistance to lower production costs and increase incomes; (iii) increased abiotic stress tolerance to improve climate change adaptation and yields on marginal, saline or drought prone soils; and (iv) facilitate a better understanding of basic phenomena such as crop evolution/domestication, ploidy, and inheritance mechanisms for understudied clonal crops.

## Results and Discussion

### Metabolomics approach – general screening

The metabolomics workflow implemented and optimized for each crop was based on a general concept (Figure 2). All plant materials collected were flash‐frozen, lyophilized, and ground to a homogenous powder before undergoing metabolite profiling workflow to ensure consistent reproducibility. A common two‐phase solvent extraction method was implemented to extract a broad range of metabolites from each type of sample. This standardized and widely used method also allowed rapid optimization of different tissue types. Furthermore, the partition into aqueous and organic phase allowed the independent analysis of polar and non‐polar extracts, which simplified sample handling, chromatographic method development, and metabolite identification. During analysis, the requirements for extraction blanks, quality controls and internal standards were implemented to maintain consistency and good laboratory practices and enable normalization and batch correction (Fernie and Klee, 2011).

### Database curation

The data generated can be deposited in public repositories addressing metabolomics in general (Metabolights, Dataverse, Metabolomics Workbench, Metexplore or Metabolonote) and/or crop specific database such as CassavaBase and MusaBase or PlantCyc. Initial fingerprinting via LC‐MS was conducted on materials to enable a rapid screen of biochemical diversity, especially focussed on secondary metabolism as this is typically where the largest proportion of chemical diversity resides (De Luca *et al.*, 2012). The bottleneck in many LC‐MS based metabolomics studies is compound identification and use of the same chromatographic method meant data generated could also be used to guide the purchase of metabolite standards for LC‐MS library generation. Typical fingerprinting screens were performed on methanol extracts and measured only one biological replicate for speed. A minimum of three biological replicates and at least two analytical platforms were used for untargeted studies, including study of both aqueous and organic extracts for more comprehensive coverage of the metabolome. For the identification of features/compounds detected during the untargeted analysis, quality controls representing a pool of samples for each species were used. Peaks detected during GC‐MS and LC‐MS analyses were identified using published libraries (e.g. NIST, GMD (Kopka *et al.*, 2005), MassBank (Horai *et al.*, 2010) etc.) and confirmed by authentic commercial standards to build a crop specific library. After database curation, automated analysis was possible for the whole dataset of each species and the identification process integrated as an element of the metabolomics data analysis pipeline. Nevertheless, manual curation was undertaken for each dataset to reduce matching errors. The analysis of isoprenoid derived metabolites, such as carotenoids and chlorophylls, was carried out using ultra high or high performance liquid chromatography coupled with a diode array detector (U/HPLC‐DAD). As the composition of leaf and tuber materials has been reported extensively (Burns *et al.*, 2003; Drapal *et al.*, 2017; Price *et al.*, 2018; Drapal *et al.*, 2019b; Drapal *et al.*, 2019c) and methods previously validated (Fraser *et al.*, 2000; Nogueira *et al.*, 2013), this was performed in a semitargeted mode in which the majority of compounds was quantified absolutely. This approach remains essential due to the intrinsic chemical nature of the photosynthetic pigments displaying a lack of amenability to MS.

### Current progress in defining the metabolome of RTB crops

**Figure 3:**
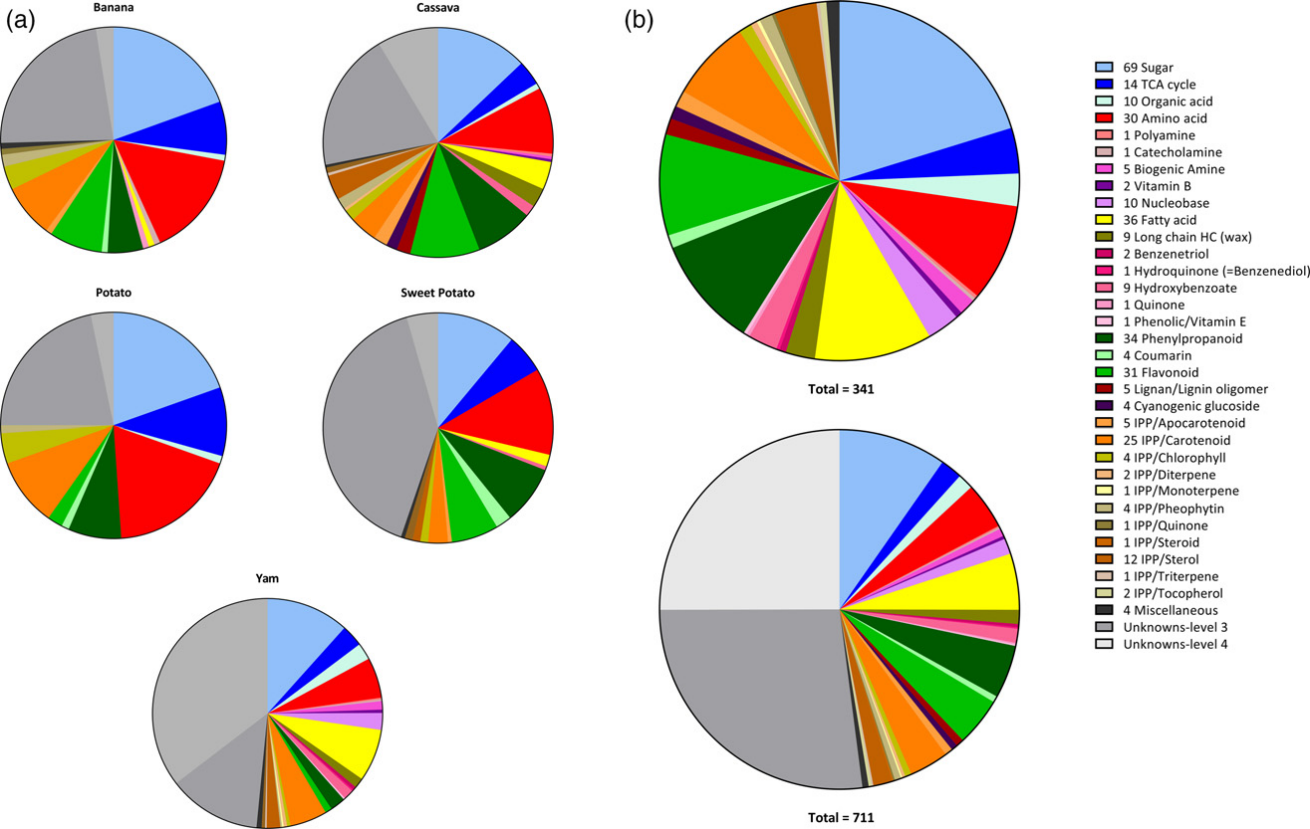
Pie‐charts showing total number of annotated compounds in RTB crops following the metabolomics workflow (Figure 2) and displayed (a) per crop and (b) for all RTB crops combined. Colours represent different compound classes and colouration follows the legend clock‐wise per each pie chart.

The database curated for banana, cassava, potato, sweet potato, and yam, currently includes over 300 identified metabolites (Table S1). Additionally, significant numbers of reoccurring unidentified features summarized as ‘unknowns’ were measured (Figure 3 and Table S2). The metabolites identified in each crop present a broad range of the plant metabolome including amino acids, organic acids, compounds of the tricarboxylic acid (TCA) cycle, isoprenoid derived compounds, phenylpropanoids, sugars, fatty acids, sterols, and corresponding subfamilies. The metabolite libraries have been implemented in the current projects of the RTB programme, facilitating the assessment of biochemical diversity, with future intentions to aid the identification of trait biomarkers in the RTB crops. The limits of metabolite concentrations have been reported to include all the available quantitative range for use in targeted breeding. This is exploitable because extremes are often favoured in crop breeding to achieve the maximum gains and enhancements above the average range and contrasts with other databases reporting the average and/or standard deviation.

Potato had the simplest biochemical profile with the presence of just 10 chemical classes (excluding unknowns); four of these related to primary metabolism. Sweet potato and banana comprised 13 and 16 chemical families, respectively, whilst the cassava and yam chemo‐libraries sum up over 20 families of compounds (Figure 3a).

Sugars was the largest annotated chemical class in all crops. This is expected in sink/storage organs as for the tissues analyzed in the collection. Similarly, chemical classes related to primary metabolism (namely amino acids, organic acids and components of the TCA cycle) were also well annotated in all species. Potato's chemical composition presented the largest proportion of these primary metabolite sectors with sugars comprising more than the other crops representing the presence of higher starch quantity.

The divergence between crop compositions resided mostly in components related to secondary metabolism. For example, yams had a greater proportion of odd‐chain fatty acids, which are rare in plants. Also characteristic of yam was the higher content and diversity of nitrogen‐containing compounds such as amines, nucleobases, and catecholamines. Nevertheless, the catecholamine dopamine was vastly more abundant (up to one order of magnitude) in *Musa*. Triterpenoids also constituted a source of chemical diversity within the RTB crops with a more complex composition found in both cassava and yam. Whilst typically these compounds were detected in the leaf tissue of the accessions, yam tubers also presented significant amounts of sterols. Crude extracts of yam presented a range of triterpenoids, including cholesterol, reflecting the production of glycosylated steroidal saponins within this crop (Sautour *et al.*, 2007). Similarly, cassava leaves showed an accumulation of amyrins and isomers, which are likely to represent the glycosylated pentacyclic saponins. High levels of β‐carotene and xanthophylls were also observed for orange‐fleshed lines of sweet potato and yam tubers, cassava roots, and *Musa* fruit, as to be expected. The largest diversity of phenolic compounds such as phenylpropanoids, coumarins, flavonoids and lignin/lignin oligomers was encountered in cassava and sweet potato, although for sweet potato many phenolics remain structurally elusive (level 3 unknown).

Unknowns comprised over half of all metabolites measured (Figure 3b) and ranged from approximately one‐quarter to one‐third of features recorded, for each individual crop following the analysis of crude extracts (Figure 3a). Distinguishing the chemical features detected via LC‐MS, and turning these into distinct compounds was challenging and will require further work to determine whether each peak is of biological origin. Given that in typical LC‐MS screening over 90% of features detected are not true metabolites (Mahieu and Patti, 2017; Aksenov *et al.*, 2017), a conservative approach to limit false positives was chosen in which only unknowns that are well characterized (e.g. via MS/MS, clear UV–vis spectra) were included in the database. The drawback to this is that the true level of unknowns may be greatly underestimated in the current database. As to be expected, the unknowns that could be assigned to a compound class were predominantly secondary metabolites (Table S2). Unknowns have been given unique identifiers to allow on‐going annotations of compounds for libraries and curation and updating of the database (Table S2).

The diversity of compound classes recorded was highest in yam and cassava, then banana, sweet potato, and lowest in potato (Figure 3a). This finding is not unsurprising, given that cassava was most intensively studied (most accessions and on all platforms) and yam is a multispecies crop and large biochemical diversity has previously been evidenced across the genus (Price *et al.*, 2016). In line with this, yam presented the highest proportion of unknowns (*c. *50%, Figure 3a); despite not undergoing LC‐MS study as per the other crops. Sweet potato also had a comparably large proportion of unknowns (*c. *45%) mostly comprising phenolic‐derived compounds, which are likely to be conjugates (Drapal *et al.*, 2019c). Accurate identification of such compounds has been shown to require comprehensive MS^3^ fragmentation and is therefore beyond that typically conducted in current metabolite screening practices (Akimoto *et al.*, 2017). Interestingly, even with the relatively extensive application of metabolomics to potato (Puzanskiy *et al.*, 2017), a large number of unknowns still exists and is mostly sugars (Table S2). Carbohydrate analysis is particularly complex, with high numbers of isomers and complex polymers that are likely to contribute to the lack of conclusive annotation. Level 3 unknowns detected in banana extracts were mostly sugars and phenolics. Furthermore, cassava had the lowest proportion of unidentified metabolites. Cassava material has been the most intensively studied area (subjected to all three analytical platforms and the largest number of tissues and accessions analyzed). This highlights that extensive analysis via diverse methods can elucidate unknowns and slowly conquer the challenge of identification, commonly touted as metabolomics’ biggest hurdle.

Overall, the observed differences between crops' metabolite databases may be the result of the application of different analytical platforms to each crop within the modular pipeline. However, current observations match that expected from literature. Dominance of particular classes of compounds in each crop reflected the plasticity of plants metabolism to develop physiological features than can be linked to particular phenotypes.

### Future developments

Presenting the ranges of metabolites recorded in a simple spreadsheet format enables the easy use of information regarding the comparative biochemical diversity of these under‐characterized crops. All compounds detected represent a portion of the steady‐state metabolome of the plant samples and can be used for untargeted data analysis to unravel the great amount of variation that can be used to guide breeding decisions. The system has proven to be robust over datasets even when measured months apart. Therefore, it is possible for future work to extend the platform from relative to proximate absolute quantification for many compounds through the generation of relative response factors to the internal standard (Cifkova *et al.*, 2012) and subsequent correction following testing of extraction recovery. Therefore, the next step will represent the transition of the untargeted pipeline to a holistic semitargeted system. From this, data can be more informative for use in flux modelling and genome‐wide reconstructions, which are essential for understanding the fundamental processes governing plant physiology (Kruger and Ratcliffe, 2015).

More elaborate sample preparations, such as solid phase extraction (SPE) and molecular recognition, via immunoaffinity, or imprinting, can be used to extend the breadth of metabolites captured and increase metabolome coverage. However, this would concurrently increase the number of unidentified compounds, which already represent a considerable proportion of the dataset (Figure 3b). Extensive structural elucidation via multistage MS fragmentation (MS^n^) and/or coupling of LC to NMR platforms (e.g. LC‐SPE‐MS/NMR) or ion mobility (e.g. LC‐IMS‐MS) has not yet become routine, largely hindered by the high capital costs at outset, and expert knowledge required for data interpretation, which is labour intensive. That said, in recent years a great deal of progress has been made towards the accessibility of tools for computational interpretation of such data (Spicer *et al.*, 2017; Tsugawa, 2018). Investments in automated structural elucidation of unidentified compounds have the potential to revolutionize metabolomics workflows by overcoming the current bottleneck of structural elucidation.

However, knowing the structure of a compound does not allow one to fully assess biological relevance. Recent years have seen a shift towards increased spatial resolution via mass spectrometry imaging and localization through cell sorting and laser microdissection etc., alongside flux‐omics and longitudinal (time‐series/developmental) applications. These applications evidence that contextualizing metabolomics data requires a detailed understanding of metabolic network dynamics and functional activity, which will become the next hurdle for the field.

Screening of complete germplasm collections will allow the establishment of a CCC that comprises the majority of biochemical diversity available. CCCs would therefore represent an advance in precision over morphological core collections and can be overlaid with genotypic collections to reduce and focus the selection on accessions with the highest prospects for successful transfer of desired traits, that is through overcoming genetic differences that do not translate through to phenotype and by encompassing biochemical traits not observed at the morphological level.

## Conclusion

### Outlook for metabolomics in breeding of RTBs

Future work appears set to capitalize on the synergy of pursuing a multiple ’omics platform for rapid progress during crop improvement and breeding. At the forefront of this pursuit is the combination of genomics and transcriptomics for breeding and trait understanding. Moreover, recently, metabolomics has been favoured to enhance precision during molecular phenotyping, and the utilization of such methods looks set to increase. Metabolomics can prove especially useful when tackling complex traits, that is those with many determinants, as the metabolome inherently reflects environmental factors and other stimuli such as chemical interactions. This is evidenced by the preference for elucidation of ‘interactomes’ such as the rhizosphere and volatile‐ome of plants by incorporating deep sequencing of the microbiome (Hu *et al.*, 2018; Jacoby and Kopriva, 2019) or atmospheric transformation of volatiles (Blande *et al.*, 2014; Li *et al*., 2016b), respectively. Combining these measurements expands the biological system to the complete local environment and therefore characterization occurs at the ecosystem level.

Improvement of RTB crops is vital for the attainment of the UN Sustainable Development Goals and improving livelihoods in the most deprived regions of the globe. In addition, the RTB crops show potential as scientific models for the analysis of complex genetic architectures, revealing the interplay between evolution and domestication in clonal crops.

Breeding and development for each of the RTB crops shows unique pitfalls and problems, yet each is widely grown due to the unique traits they present. The complexities that have hindered crop improvement and agronomic development for production of RTBs to date may also be the crops’ largest saviours. In light of climate change, the large morphological plasticity, limited genetic assimilation, and resilience of these crops to extreme conditions and low technology agricultural systems provide the potential to adapt and overcome the impacts of global warming and, therefore, provide the incentive to increase research efforts towards these critically important understudied RTB crops. To ensure this, the breeding community needs to move beyond viewing metabolomics and other ’omics as a hypothesis‐free service science to techniques that can be integrated to solve complex biological questions in a rapid, large‐scale manner. Ironically, the initial characterization of plant genetic resources and diversity available is crucial to pose the biological questions for investigation and, as such, metabolomics can progress on both fronts.

## Experimental procedures

Samples from *in vitro* cultures and plants grown in the field were harvested, flash‐frozen with liquid nitrogen, and lyophilized to remove all water content. The samples comprised a collection of different tissues, for example leaf, root, tuber, stem, and fruit from each crop. The tissue samples were then ground to a fine powder and metabolites extracted. Sample preparation and extraction and the profiling procedure of the extracts was based on previously published protocols and optimized for each crop to account for the matrix effects of the respective tissue (Perez‐Fons *et al.*, 2014; Price *et al.*, 2016; Drapal *et al.*, 2017; Price *et al.*, 2017; Price *et al.*, 2018; Drapal *et al.*, 2019a; Drapal *et al.*, 2019b; Drapal *et al.*, 2019c). To account for the difference in chemical properties of the metabolites, three different platforms were utilized in a modular manner for the screening process: ultra/high performance liquid chromatography with diode array detector (U/HPLC‐DAD), liquid chromatography‐mass spectrometry (LC‐MS) and gas chromatography‐mass spectrometry (GC‐MS). The yam materials underwent GC‐MS of both polar and non‐polar extracts alongside HPLC‐DAD of the non‐polar phase. All other crops underwent GC‐MS and LC‐MS analysis on polar extracts and UPLC‐DAD of non‐polar extracts. Non‐polar extracts from cassava and sweet potato were also subjected to GC‐MS analysis.

The curation of crop specific libraries with identified metabolites followed the same workflow for both the GC‐MS and LC‐MS analytical platforms (Figure 3), whereas an established UPLC‐DAD library was used for all crops (Fraser *et al.*, 2000; Burns *et al.*, 2003) with an extended version used for yam and sweet potato (Price *et al.*, 2018). All features detected in the generated sample set were aligned and following statistical analysis, significant features were identified and confirmed with standards (Fernie and Klee, 2011). GC‐MS data were processed via AMDIS (v2.71, NIST) whereas the alignment and filtering of chromatograms for LC‐MS was achieved via metaMS (Wehrens *et al.*, 2014; Franceschi *et al.*, 2014). U/HPLC‐PDA data were analyzed via Empower 2^TM^ software (Waters Corp.). Manual confirmation of the identified compounds was carried out (Table S1) and recurrent unidentified features that represent hypothetical compounds have been reported with unique identifiers per species (Table S2) (Bino *et al.*, 2004). Normalization to internal standards and sample weight allowed relative quantification, concatenation of data from the platforms, and subsequent comparison between tissue types and species. For the UPLC, absolute quantification for the major photosynthetic compounds (β‐carotene, violaxanthin, neoxanthin, phytoene, phytofluene, chlorophyll *a*, chlorophyll *b*, β‐cryptoxanthin, lutein, antheraxanthin, and zeaxanthin) was achieved via comparison with dose–response curves of authentic commercially available standards. For carotenoids, for which an authentic standard was not available, quantification was based on standard curves of carotenoids with the closest chemical structure and spectral properties similarity. When compounds were detected on more than one analytical platform, the values reported in the database represent that of the maxima recorded and the analytical technique that proved to be more amenable was cited first. The database and pie‐charts were created in Microsoft Excel 2013.

As the compiled dataset was comprised of numerous independent analyses undertaken over a three‐year time‐frame, the metabolite ranges reported for each crop differed in the number of samples analyzed and replicate measurements made. However, for each metabolite reported per crop a minimum of 12 measurements were taken and the validity and repeatability of measures were controlled within each independent study. Furthermore, analytical drift and different response factors were controlled platform‐to‐platform, batch–to‐batch and study‐to‐study via the analysis of both reference sample (quality control) and reference metabolite (internal standard) to ensure robustness.

## Conflicts of interest

The authors declare that they have no conflicts of interest in accordance with journal policy.

## Author contributions

EP, MD and LP‐F generated the datasets, assembled the figures, compiled supplementary tables, and drafted the manuscript and devised the concept. DA, RB, BH, MR and RS selected plant materials, aided interpretation of results, and elaborated the manuscript. LABL‐L selected plant materials, aided interpretation of results, coordinated across centres, and elaborated the manuscript. PDF aided interpretation of results, drafted and edited the manuscript, secured funding and devised the concept.

## Supporting information


**Table S1.** Database of metabolite concentration range per crop.

**Table S2.** Lists of recurrent unknowns identified per crop.

## Data Availability

All data compiled for this resource paper are included in this published article (and its supplementary information files) and references to the original publications/data sets are cited.

## References

[tpj14649-bib-0001] Akimoto, N. , Ara, T. , Nakajima, D. ***et al*** **.** (2017) FlavonoidSearch: a system for comprehensive flavonoid annotation by mass spectrometry. Sci. Rep. 7, 1–9. 28455528 10.1038/s41598-017-01390-3 PMC5430893

[tpj14649-bib-0002] Aksenov, A.A. , Silva, R.Da , Knight, R. , Lopes, N.P. and Dorrestein, P.C. (2017) Global chemical analysis of biology by mass spectrometry. Nat. Rev. Chem. 1, 1–20.

[tpj14649-bib-0003] Basu, S. , Duren, W. , Evans, C.R. , Burant, C.F. , Michailidis, G. and Karnovsky, A. (2017) Sparse network modeling and metscape‐based visualization methods for the analysis of large‐scale metabolomics data. Bioinformatics, 33, 1545–1553. 28137712 10.1093/bioinformatics/btx012 PMC5860222

[tpj14649-bib-0004] Bino, R.J. , Hall, R.D. , Fiehn, O. ***et al*** **.** (2004) Potential of metabolomics as a functional genomics tool. Trends Plant Sci. 9, 418–425. 15337491 10.1016/j.tplants.2004.07.004

[tpj14649-bib-0005] Blande, J.D. , Holopainenen, J.K. and Niinemets, Ü. (2014) Plant volatiles in polluted atmospheres: stress responses and signal degradation. Plant. Cell Environ. 37, 1892–1904. 24738697 10.1111/pce.12352 PMC4289706

[tpj14649-bib-0006] Bredeson, J.V. , Lyons, J.B. , Prochnik, S.E. ***et al*** **.** (2016) Sequencing wild and cultivated cassava and related species reveals extensive interspecific hybridization and genetic diversity. Nat. Biotechnol. 34, 562–570. 27088722 10.1038/nbt.3535

[tpj14649-bib-0007] Burns, J. , Fraser, P.D. and Bramley, P.M. (2003) Identification and quantification of carotenoids, tocopherols and chlorophylls in commonly consumed fruits and vegetables. Phytochemistry, 62, 939–947. 12590121 10.1016/s0031-9422(02)00710-0

[tpj14649-bib-0008] Castañeda‐Álvarez, N.P. , Khoury, C.K. , Achicanoy, H.A. ***et al*** **.** (2016) Global conservation priorities for crop wild relatives. Nat. Plants, 2, 16022. 27249561 10.1038/nplants.2016.22

[tpj14649-bib-0009] Cenci, A. , Hueber, Y. , Zorrilla‐Fontanesi, Y. ***et al*** **.** (2019) Effect of paleopolyploidy and allopolyploidy on gene expression in banana. BMC Genom. 20, 1–12. 10.1186/s12864-019-5618-0 PMC6438041 30917780

[tpj14649-bib-0010] Chan, E.K.F. , Rowe, H.C. , Hansen, B.G. and Kliebenstein, D.J. (2010) The complex genetic architecture of the metabolome. PLoS Genet. 6, e1001198. 21079692 10.1371/journal.pgen.1001198 PMC2973833

[tpj14649-bib-0011] Christelová, P. , Langhe, E.De , Hřibová, E. ***et al*** **.** (2017) Molecular and cytological characterization of the global Musa germplasm collection provides insights into the treasure of banana diversity. Biodivers. Conserv. 26, 801–824.

[tpj14649-bib-0012] Cifkova, E. , Holcapek, M. , Lisa, M. , Ovcacikova, M. , Lycka, A. , Lynen, F. and Sandra, P. (2012) Nontargeted quantitation of lipid classes using hydrophilic interaction liquid chromatography‐electrospray ionization mass spectrometry with single internal standard and response factor approach. Anal. Chem. 84, 10064–10070. 23072569 10.1021/ac3024476

[tpj14649-bib-0013] D'Hont, A. , Denoeud, F. , Aury, J.‐M.J.‐M. ***et al*** **.** (2012) The banana (Musa acuminata) genome and the evolution of monocotyledonous plants. Nature, 488, 213–218. 22801500 10.1038/nature11241

[tpj14649-bib-0014] Daygon, V.D. , Calingacion, M. , Forster, L.C. ***et al*** **.** (2017) Metabolomics and genomics combine to unravel the pathway for the presence of fragrance in rice. Sci. Rep. 7, 1–12. 28821745 10.1038/s41598-017-07693-9 PMC5562744

[tpj14649-bib-0015] Dépigny, S. , Tchotang, F. , Talla, M. , Fofack, D. , Essomé, D. , Ebongué, J.P. , Kengni, B. and Lescot, T. (2018) The ‘Plantain‐Optim’ dataset: Agronomic traits of 405 plantains every 15 days from planting to harvest. Data Br. 17, 671–680. 10.1016/j.dib.2018.01.065 PMC5852287 29552618

[tpj14649-bib-0016] Drapal, M. , Farfan‐Vignolo, E.R. , Gutierrez, O.R. , Bonierbale, M. , Mihovilovich, E. and Fraser, P.D. (2017) Identification of metabolites associated with water stress responses in Solanum tuberosum L. clones. Phytochemistry, 135, 24–33. 27964835 10.1016/j.phytochem.2016.12.003

[tpj14649-bib-0017] Drapal, M. , Carvalho, E. , Ovalle Rivera, T.M. , Becerra Lopez‐Lavalle, L.A. and Fraser, P.D. (2019a) Capturing biochemical diversity in Cassava ( Manihot esculenta Crantz) through the application of metabolite profiling. J. Agric. Food Chem. 67, 986–993. 30557498 10.1021/acs.jafc.8b04769 PMC6346375

[tpj14649-bib-0018] Drapal, M. , de Carvalho, E.B. , Rouard, M. ***et al*** **.** (2019b) Metabolite profiling characterises chemotypes of Musa diploids and triploids at juvenile and pre‐flowering growth stages. Sci. Rep. 9, 4657. 30874619 10.1038/s41598-019-41037-z PMC6420674

[tpj14649-bib-0019] Drapal, M. , Rossel, G. , Heider, B. and Fraser, P.D. (2019c) Metabolic diversity in sweet potato (Ipomoea batatas, Lam.) leaves and storage roots. Hortic. Res. 6, 2. 30603089 10.1038/s41438-018-0075-5 PMC6312539

[tpj14649-bib-0020] Ellis, D. , Chavez, O. , Coombs, J. , Soto, J. , Gomez, R. , Douches, D. , Panta, A. , Silvestre, R. and Anglin, N.L. (2018) Genetic identity in genebanks: application of the SolCAP 12K SNP array in fingerprinting and diversity analysis in the global in trust potato collection. Genome, 61, 523–537. 29792822 10.1139/gen-2017-0201

[tpj14649-bib-0021] Fernie, A.R. and Klee, H.J. (2011) The use of natural genetic diversity in the understanding of metabolic organization and regulation. Front. Plant Sci. 2, 59. 22645543 10.3389/fpls.2011.00059 PMC3355787

[tpj14649-bib-0022] Fernie, A.R. and Schauer, N. (2009) Metabolomics‐assisted breeding: a viable option for crop improvement? Trends Genet. 25, 39–48. 19027981 10.1016/j.tig.2008.10.010

[tpj14649-bib-0023] Food and Agriculture Organization of the United Nations . (2019) FAOSTAT Database. Available at: http://www.fao.org/faostat [Accessed July 7, 2019].

[tpj14649-bib-0024] Franceschi, P. , Mylonas, R. , Shahaf, N. ***et al*** **.** (2014) MetaDB a data processing workflow in untargeted MS‐based metabolomics experiments. Front. Bioeng. Biotechnol. 2, 1–12. 25566535 10.3389/fbioe.2014.00072 PMC4267269

[tpj14649-bib-0025] Fraser, P.D. , Pinto, M.E.S. , Holloway, D.E. and Bramley, P.M. (2000) Application of high‐performance liquid chromatography with photodiode array detection to the metabolic profiling of plant isoprenoids. Plant J. 24, 551–558. 11115136 10.1046/j.1365-313x.2000.00896.x

[tpj14649-bib-0026] Friedmann, M. , Asfaw, A. , Anglin, N. ***et al*** **.** (2018) Genomics‐Assisted Breeding in the CGIAR Research Program on Roots, Tubers and Bananas (RTB). Agriculture, 8, 89.

[tpj14649-bib-0027] Girma, G. , Korie, S. , Dumet, D. and Franco, J. (2012) Improvement of accession distinctiveness as an added value to the global worth of the Yam (Dioscorea spp) Genebank. Int. J. Conserv. Sci. 3, 199–206.

[tpj14649-bib-0028] Girma, G. , Bhattacharjee, R. , Lopez‐Montes, A. , Gueye, B. , Ofodile, S. , Franco, J. and Abberton, M. (2018) Re‐defining the yam (Dioscorea spp.) core collection using morphological traits. Plant Genet. Resour. Charact. Util. 16, 193–200.

[tpj14649-bib-0029] Grennan, A.K. (2009) MoTo DB: a metabolic database for tomato. Plant Physiol. 151, 1701–1702. 19965978 10.1104/pp.109.900308 PMC2785969

[tpj14649-bib-0030] Hardigan, M.A. , Laimbeer, F.P.E. , Newton, L. ***et al*.** (2017) Genome diversity of tuber-bearing Solanum uncovers complex evolutionary history and targets of domestication in the cultivated potato. Proc. Natl. Acad. Sci. USA, 114, E9999–E10008. 29087343 10.1073/pnas.1714380114 PMC5699086

[tpj14649-bib-0031] Horai, H. , Arita, M. , Kanaya, S. ***et al*** **.** (2010) MassBank: a public repository for sharing mass spectral data for life sciences. J. Mass Spectrom. 45, 703–714. 20623627 10.1002/jms.1777

[tpj14649-bib-0032] Hu, L. , Robert, C.A.M. , Cadot, S. ***et al*** **.** (2018) Root exudate metabolites drive plant‐soil feedbacks on growth and defense by shaping the rhizosphere microbiota. Nat. Commun. 9, 1–13. 30013066 10.1038/s41467-018-05122-7 PMC6048113

[tpj14649-bib-0033] Huamán, Z. , Ortiz, R. and Gómez, R. (2000) Selecting a Solanum tuberosum subsp. andigena core collection using morphological, geographical, disease and pest descriptors. Am. J. Potato Res. 77, 183–190.

[tpj14649-bib-0034] Jacoby, R.P. and Kopriva, S. (2019) Metabolic niches in the rhizosphere microbiome: new tools and approaches to analyse metabolic mechanisms of plant–microbe nutrient exchange. J. Exp. Bot. 70, 1087–1094. 30576534 10.1093/jxb/ery438

[tpj14649-bib-0035] Jansky, S.H. , Dawson, J. and Spooner, D.M. (2015) How do we address the disconnect between genetic and morphological diversity in germplasm collections? Am. J. Bot. 102, 1213–1215. 26290545 10.3732/ajb.1500203

[tpj14649-bib-0036] Kennedy, G. , Raneri, J.E. , Stoian, D. ***et al*** **.** (2019) Roots, tubers and bananas: contributions to food security In Encyclopedia of Food Security and Sustainability (Ferranti P., Berry E.M. and Anderson J.R. eds.). Amsterdam, Netherlands; Oxford, UK; Cambridge, MA: Elsevier, pp. 231–256.

[tpj14649-bib-0037] Kim, J.‐H. , Chung, I.K. and Kim, K.‐M. (2017) Construction of a genetic map using EST‐SSR markers and QTL analysis of major agronomic characters in hexaploid sweet potato (Ipomoea batatas (L.) Lam). PLoS ONE, 12, e0185073. 29020092 10.1371/journal.pone.0185073 PMC5636084

[tpj14649-bib-0038] Kopka, J. , Schauer, N. , Krueger, S. ***et al*** **.** (2005) GMD@CSB.DB: the Golm metabolome database. Bioinformatics, 21, 1635–1638. 15613389 10.1093/bioinformatics/bti236

[tpj14649-bib-0039] Kruger, N.J. and Ratcliffe, R.G. (2015) Fluxes through plant metabolic networks: measurements, predictions, insights and challenges. Biochem. J. 465, 27–38. 25631681 10.1042/BJ20140984

[tpj14649-bib-0040] Kumar, R. , Bohra, A. , Pandey, A.K. , Pandey, M.K. and Kumar, A. (2017) Metabolomics for Plant Improvement: Status and Prospects. Front. Plant Sci. 8, 1–27. 28824660 10.3389/fpls.2017.01302 PMC5545584

[tpj14649-bib-0041] Kundapura Venkataramana, R. , Hastantram Sampangi‐Ramaiah, M. , Ajitha, R. , N. Khadke, G. and Chellam, V. (2015) Insights into Musa balbisiana and Musa acuminata species divergence and development of genic microsatellites by transcriptomics approach. Plant Gene, 4, 78–82.

[tpj14649-bib-0042] Li, B. , Zhang, Y. , Mohammadi, S.A. , Huai, D. , Zhou, Y. and Kliebenstein, D.J. (2016a) An integrative genetic study of rice metabolism, growth and stochastic variation reveals potential C/N partitioning loci. Sci. Rep. 6, 1–13. 27440503 10.1038/srep30143 PMC4954952

[tpj14649-bib-0043] Li, T. , Blande, J.D. and Holopainen, J.K. (2016b) Atmospheric transformation of plant volatiles disrupts host plant finding. Sci. Rep. 6, 33851. 27651113 10.1038/srep33851 PMC5030639

[tpj14649-bib-0044] Li, M. , Yang, S. , Xu, W. ***et al*** **.** (2019) The wild sweetpotato (Ipomoea trifida) genome provides insights into storage root development. BMC Plant Biol. 19, 119. 30935381 10.1186/s12870-019-1708-z PMC6444543

[tpj14649-bib-0045] Luca, V.De , Salim, V. , Atsumi, S.M. ***et al*** **.** (2012) Mining the biodiversity of plants: a revolution in the making. Science, 336, 1658–1661. 22745417 10.1126/science.1217410

[tpj14649-bib-0046] Mahieu, N.G. and Patti, G.J. (2017) Systems‐level annotation of a metabolomics data set reduces 25 000 features to fewer than 1000 unique metabolites. Anal. Chem. 89, 10397–10406. 28914531 10.1021/acs.analchem.7b02380 PMC6427824

[tpj14649-bib-0047] Misra, B.B. , Langefeld, C. , Olivier, M. and Cox, L.A. (2019) Integrated omics: tools, advances and future approaches. J. Mol. Endocrinol. R21–R45. 10.1530/JME-18-0055 30006342

[tpj14649-bib-0048] Monden, Y. and Tahara, M. (2017) Genetic linkage analysis using DNA markers in sweetpotato. Breed. Sci. 67, 41–51. 28465667 10.1270/jsbbs.16142 PMC5407921

[tpj14649-bib-0049] Muñoz‐Rodríguez, P. , Carruthers, T. , Wood, J.R.I. ***et al*** **.** (2018) Reconciling conflicting phylogenies in the origin of sweet potato and dispersal to polynesia. Curr. Biol. 28, 1246–1256.e12. 29657119 10.1016/j.cub.2018.03.020

[tpj14649-bib-0050] Němečková, A. , Christelová, P. , Čížková, J. ***et al*** **.** (2018) Molecular and cytogenetic study of East African highland banana. Front. Plant Sci. 9, 1–13. 30337933 10.3389/fpls.2018.01371 PMC6180188

[tpj14649-bib-0051] Nogueira, M. , Mora, L. , Enfissi, E.M.A. , Bramley, P.M. and Fraser, P.D. (2013) Subchromoplast sequestration of carotenoids affects regulatory mechanisms in tomato lines expressing different carotenoid gene combinations. Plant Cell, 25, 4560–4579. 24249831 10.1105/tpc.113.116210 PMC3875736

[tpj14649-bib-0052] Nyine, M. , Uwimana, B. , Swennen, R. , Batte, M. , Brown, A. , Christelová, P. , Hřibová, E. , Lorenzen, J. and Doleziel, J. (2017) Trait variation and genetic diversity in a banana genomic selection training population. PLoS ONE, 12, 1–23. 10.1371/journal.pone.0178734 PMC5460855 28586365

[tpj14649-bib-0053] Oliveira, E.J. , Filho, O.S.O. and Santos, V.S. (2015) Classification of cassava genotypes based on qualitative and quantitative data. Genet. Mol. Res. 14, 906–924. 25730029 10.4238/2015.February.2.14

[tpj14649-bib-0054] Perez‐Fons, L. , Wells, T. , Corol, D.I. , Ward, J.L. , Gerrish, C. , Beale, M.H. , Seymour, G.B. , Bramley, P.M. and Fraser, P.D. (2014) A genome‐wide metabolomic resource for tomato fruit from Solanum pennellii. Sci. Rep. 4, 3859. 24457419 10.1038/srep03859 PMC3900926

[tpj14649-bib-0055] Pinu, F.R. , Beale, D.J. , Paten, A.M. , Kouremenos, K. , Swarup, S. , Schirra, H.J. and Wishart, D. (2019) Systems biology and multi‐omics integration: viewpoints from the metabolomics research community. Metabolites, 9, 76. 10.3390/metabo9040076 PMC6523452 31003499

[tpj14649-bib-0056] Price, E.J. , Wilkin, P. , Sarasan, V. and Fraser, P.D. (2016) Metabolite profiling of Dioscorea (yam) species reveals underutilised biodiversity and renewable sources for high‐value compounds. Sci. Rep. 6, 29136. 27385275 10.1038/srep29136 PMC4935876

[tpj14649-bib-0057] Price, E.J. , Bhattacharjee, R. , Lopez‐Montes, A. and Fraser, P.D. (2017) Metabolite profiling of yam (Dioscorea spp.) accessions for use in crop improvement programmes. Metabolomics, 13, 144. 29104519 10.1007/s11306-017-1279-7 PMC5641283

[tpj14649-bib-0058] Price, E.J. , Bhattacharjee, R. , Lopez‐Montes, A. and Fraser, P.D. (2018) Carotenoid profiling of yams: clarity, comparisons and diversity. Food Chem. 259, 130–138. 29680035 10.1016/j.foodchem.2018.03.066

[tpj14649-bib-0059] Puzanskiy, R.K. , Yemelyanov, V.V. , Gavrilenko, T.A. and Shishova, M.F. (2017) The perspectives of metabolomic studies of potato plants. Russ. J. Genet. Appl. Res. 7, 744–756.

[tpj14649-bib-0060] Rahajeng, W. and Rahayuningsih, S.A. (2017) Agronomic performance, variance component, and diversity of sixty‐two sweet potato accessions. Biodiversitas. J. Biol. Divers. 18, 95–100.

[tpj14649-bib-0061] Riedelsheimer, C. , Czedik‐Eysenberg, A. , Grieder, C. ***et al*** **.** (2012) Genomic and metabolic prediction of complex heterotic traits in hybrid maize. Nat. Genet. 44, 217–220. 22246502 10.1038/ng.1033

[tpj14649-bib-0062] Sanginga, N. (2015) Root and tuber crops (cassava, yam, potato and sweet potato) In Feeding Africa: An Action Plan for African Agricultural Transformation. Duhar, Senegal: African Development Bank Group, pp. 1–26.

[tpj14649-bib-0063] Sarah, G. , Homa, F. , Pointet, S. ***et al*** **.** (2017) A large set of 26 new reference transcriptomes dedicated to comparative population genomics in crops and wild relatives. Mol. Ecol. Resour. 17, 565–580. 27487989 10.1111/1755-0998.12587

[tpj14649-bib-0064] Sautour, M. , Mitaine‐Offer, A.‐C. and Lacaille‐Dubois, M.‐A. (2007) The Dioscorea genus: a review of bioactive steroid saponins. J. Nat. Med. 61, 91–101.

[tpj14649-bib-0065] Scarcelli, N. , Chaïr, H. , Causse, S. , Vesta, R. , Couvreur, T.L.P. and Vigouroux, Y. (2017) Crop wild relative conservation: wild yams are not that wild. Biol. Conserv. 210, 325–333.

[tpj14649-bib-0066] Schwahn, K. , de Souza, L.P. , Fernie, A.R. and Tohge, T. (2014) Metabolomics‐assisted refinement of the pathways of steroidal glycoalkaloid biosynthesis in the tomato clade. J. Integr. Plant Biol. 56, 864–875. 25109688 10.1111/jipb.12274

[tpj14649-bib-0067] Scott, G.J. , Rosegrant, M.W. and Ringler, C. (2000) Roots and tubers for the 21st century: trends, projections and policy options, 2020 vision discussion papers 31. Washington, D.C.: International Food Policy Research Institute (IFPRI).

[tpj14649-bib-0068] Shannon, P. (2003) Cytoscape: a software environment for integrated models of biomolecular interaction networks. Genome Res. 13, 2498–2504. 14597658 10.1101/gr.1239303 PMC403769

[tpj14649-bib-0069] Sharma, S.K. and Bryan, G.J. (2017) Genome sequence‐based marker development and genotyping in potato. The Potato Genome. 307–326.

[tpj14649-bib-0070] Spicer, R. , Salek, R.M. , Moreno, P. , Cañueto, D. and Steinbeck, C. (2017) Navigating freely‐available software tools for metabolomics analysis. Metabolomics, 13, 1–16. 28890673 10.1007/s11306-017-1242-7 PMC5550549

[tpj14649-bib-0071] Sprenger, H. , Erban, A. , Seddig, S. ***et al*** **.** (2018) Metabolite and transcript markers for the prediction of potato drought tolerance. Plant Biotechnol. J. 16, 939–950. 28929574 10.1111/pbi.12840 PMC5866952

[tpj14649-bib-0072] Tamiru, M. , Natsume, S. , Takagi, H ***et al*** **.** (2017) Genome sequencing of the staple food crop white Guinea yam enables the development of a molecular marker for sex determination. BMC Biol. 15, 86. 28927400 10.1186/s12915-017-0419-x PMC5604175

[tpj14649-bib-0073] Tay, D. (2013) Tropical and subtropical root and tuber crops InConservation of Tropical Plant Species (Normah M.N., Chin H.F. and Reed B.M. eds.). New York, NY: Springer, New York, pp. 249–292.

[tpj14649-bib-0074] Tsugawa, H. (2018) Advances in computational metabolomics and databases deepen the understanding of metabolisms. Curr. Opin. Biotechnol. 54, 10–17. 29413746 10.1016/j.copbio.2018.01.008

[tpj14649-bib-0076] Wang, W. , Feng, B. , Xiao, J. ***et al*** **.** (2014) Cassava genome from a wild ancestor to cultivated varieties. Nat. Commun. 5, 5110. 25300236 10.1038/ncomms6110 PMC4214410

[tpj14649-bib-0077] Ward, J. , Rakszegi, M. , Bedő, Z. , Shewry, P.R. and Mackay, I. (2015) Differentially penalized regression to predict agronomic traits from metabolites and markers in wheat. BMC Genet. 16, 19. 25879431 10.1186/s12863-015-0169-0 PMC4348103

[tpj14649-bib-0078] Wehrens, R. , Weingart, G. and Mattivi, F. (2014) metaMS: an open‐source pipeline for GC–MS‐based untargeted metabolomics. J. Chromatogr. B, 966, 109–116. 10.1016/j.jchromb.2014.02.051 24656939

[tpj14649-bib-0079] Wei, J. , Wang, A. , Li, R. , Qu, H. and Jia, Z. (2018) Metabolome‐wide association studies for agronomic traits of rice. Heredity (Edinb), 120, 342–355. 29225351 10.1038/s41437-017-0032-3 PMC5842221

[tpj14649-bib-0080] van Wesemael, J. , Kissel, E. , Eyland, D. , Lawson, T. , Swennen, R. and Carpentier, S. (2019) Using growth and transpiration phenotyping under controlled conditions to select water efficient banana genotypes. Front. Plant Sci. 10, 1–14. 30972089 10.3389/fpls.2019.00352 PMC6443892

[tpj14649-bib-0081] Wolfe, M.D. , Kulakow, P. , Rabbi, I.Y. and Jannink, J.‐L. (2016) Marker‐based estimates reveal significant nonadditive effects in clonally propagated Cassava (Manihot esculenta): implications for the prediction of total genetic value and the selection of varieties. G3&amp;#58; Genes|Genomes|Genetics, 6, 3497–3506. 27587297 10.1534/g3.116.033332 PMC5100848

[tpj14649-bib-0082] Xu, Y. (2016) Envirotyping for deciphering environmental impacts on crop plants. Theor. Appl. Genet. 129, 653–673. 26932121 10.1007/s00122-016-2691-5 PMC4799247

[tpj14649-bib-0083] Xu, X. , Pan, S. , Cheng, S. ***et al*** **.** (2011) Genome sequence and analysis of the tuber crop potato. Nature, 475, 189–195. 21743474 10.1038/nature10158

[tpj14649-bib-0084] Yang, J. , Moeinzadeh, M.‐H. , Kuhl, H. ***et al*** **.** (2017) Haplotype‐resolved sweet potato genome traces back its hexaploidization history. Nat. Plants, 3, 696–703. 28827752 10.1038/s41477-017-0002-z

[tpj14649-bib-0085] Zivy, M. , Wienkoop, S. , Renaut, J. , Pinheiro, C. , Goulas, E. and Carpentier, S. (2015) The quest for tolerant varieties: the importance of integrating ‘omics’ techniques to phenotyping. Front. Plant Sci. 6, 1–11. 26217344 10.3389/fpls.2015.00448 PMC4496562

