# Supplementary material for: Metabolite database for root, tuber, and banana crops to facilitate modern breeding in understudied crops
Source: Plant J. 2020 Jan 22;101(6):1258–68. doi: 10.1111/tpj.14649 (PMC7383867; doi:10.1111/tpj.14649)
Supplement: Supplementary file 3 [file TPJ-101-1258-s003.docx]

Supplementary table ST1. Database of metabolite concentration range per crop.

Supplementary table ST2. Lists of recurrent unknowns identified per crop.
